# Multidisciplinary approach detects speciation within the kissing bug *Panstrongylus rufotuberculatus* populations (Hemiptera, Heteroptera, Reduviidae)

**DOI:** 10.1590/0074-02760210259

**Published:** 2022-02-02

**Authors:** Sebastián Pita, Andrés Gómez-Palacio, Pedro Lorite, Jean Pierre Dujardin, Tamara Chavez, Anita G Villacís, Cleber Galvão, Yanina Panzera, Lucía Calleros, Santiago Pereyra-Mello, Gabriela Burgueño-Rodríguez, Francisco Panzera

**Affiliations:** 1 Universidad de la República, Facultad de Ciencias, Sección Genética Evolutiva, Montevideo, Uruguay; 2 Universidad Pedagógica y Tecnológica de Colombia, Laboratorio de Investigación en Genética Evolutiva, Boyacá, Colombia; 3 Universidad de Jaén, Departamento de Biología Experimental, Área de Genética, Jaén, Spain; 4 University of Montpellier, Institut de Recherche pour le Développement, International Campus in Baillarguet, Montpellier, Occitanie, France; 5 Instituto Nacional de Laboratorios de Salud, Laboratorio de Entomología Médica, La Paz, Bolivia; 6 Pontificia Universidad Católica del Ecuador, Facultad de Ciencias Exactas y Naturales, Escuela de Ciencias Biológicas, Centro de Investigación para la Salud en América Latina, Nayón, Quito, Ecuador; 7 Fundação Oswaldo Cruz-Fiocruz, Instituto Oswaldo Cruz, Laboratório Nacional e Internacional de Referência em Taxonomia de Triatomíneos, Rio de Janeiro, RJ, Brasil; 8 Universidad de la República, Centro Universitario Regional, Departamento de Ciencias Biológicas, Laboratorio de Genética Molecular Humana, Salto, Uruguay

**Keywords:** Chagas disease vectors, cytochrome b gene, cytochrome C oxidase subunit I, internal transcribed spacer 2, karyotype evolution, morphometric analyses, sex chromosome fusion

## Abstract

**BACKGROUND:**

*Panstrongylus rufotuberculatus* (Hemiptera-Reduviidae) is a triatomine species with a wide geographic distribution and a broad phenotypic variability. In some countries, this species is found infesting and colonising domiciliary ecotopes representing an epidemiological risk factor as a vector of *Trypanosoma cruzi*, etiological agent of Chagas disease. In spite of this, little is known about *P. rufotuberculatus* genetic diversity.

**METHODS:**

Cytogenetic studies and DNA sequence analyses of one nuclear (ITS-2) and two mitochondrial DNA sequences (*cyt b* and *coI*) were carried out in *P. rufotuberculatus* individuals collected in Bolivia, Colombia, Ecuador and Mexico. Moreover, a geometric morphometrics study was applied to Bolivian, Colombian, Ecuadorian and French Guiana samples.

**OBJECTIVES:**

To explore the genetic and phenetic diversity of *P. rufotuberculatus* from different countries, combining chromosomal studies, DNA sequence analyses and geometric morphometric comparisons.

**FINDINGS:**

We found two chromosomal groups differentiated by the number of X chromosomes and the chromosomal position of the ribosomal DNA clusters. In concordance, two main morphometric profiles were detected, clearly separating the Bolivian sample from the other ones. Phylogenetic DNA analyses showed that both chromosomal groups were closely related to each other and clearly separated from the remaining *Panstrongylus* species. High nucleotide divergence of *cyt b* and *coI* fragments were observed among *P. rufotuberculatus* samples from Bolivia, Colombia, Ecuador and Mexico (Kimura 2-parameter distances higher than 9%).

**MAIN CONCLUSIONS:**

Chromosomal and molecular analyses supported that the two chromosomal groups could represent different closely related species. We propose that Bolivian individuals constitute a new *Panstrongylus* species, being necessary a detailed morphological study for its formal description. The clear morphometric discrimination based on the wing venation pattern suggests such morphological description might be conclusive.

The Triatominae subfamily (Hemiptera: Heteroptera: Reduviidae) includes more than 150 blood-sucking species grouped into 16 genera.^1^ ^,^ ^2^ These insects act as vectors of *Trypanosoma cruzi*, the etiological agent of Chagas disease, which is recognised as the most serious human parasitic disease of Latin America, affecting 5-6 million people.^2^ ^,^ ^3^ In the absence of vaccines or adequate drugs for large-scale treatment, the reduction of disease incidence depends mainly on vector control of bug’s populations in human dwellings.^2^ ^,^ ^3^ An accurate taxonomic identification and knowledge about the genetics of these insects are keys to ensure successful entomological surveillance after control campaigns.^2^

The *Panstrongylus* genus belongs to the Triatomini tribe and is included within the North American lineage, constituting a paraphyletic group.^4^ ^,^ ^5^ ^,^ ^6^ *Panstrongylus* is the third most relevant genus within Triatominae in terms of species richness (one fossil and 14 living species) and epidemiological impact.^7^ Among these species, *Panstrongylus megistus* is the most significant Chagas disease vector, particularly in Brazil. Although the remaining *Panstrongylus* species are primarily sylvatic, some of them have gained attention, as they are involved in domiciliation processes and hence the transmission of Chagas disease to humans. *Panstrongylus rufotuberculatus* is one of these species, with a wide distribution area extending from Mexico to Argentina, from lowland rainforests to arid highlands of up to 2600m above sea level.^1^ ^,^ ^7^ ^,^ ^8^ Additionally it is adapted to dry as well as humid ecotopes, being found in a great variety of sylvatic hosts including armadillos, kinkajous, opossums, rodents, bats and birds burrows.^1^ Furthermore, several authors reported breeding colonies of *P. rufotuberculatus* inside and around dwellings in Bolivia,^8^ ^,^ ^9^ ^,^ ^10^ Colombia,^11^ Ecuador,^12^ ^,^ ^13^ Peru^14^ and Venezuela.^15^ Some studies highlight the high rates of house infestation^10^ ^,^ ^11^ and infection with *Trypanosoma cruzi*.^12^ The domiciliary presence of *P. rufotuberculatus* represents an important epidemiological risk factor for Chagas disease transmission in several Latin American countries, mainly in areas where the principal vector (e.g. *Triatoma infestans*) is absent due to vector control programs.^10^

Following the description of the male holotype from Panama by Champion (1899), several authors have emphasised the chromatic, morphologic and morphometric variation of *P. rufotuberculatus* along their geographical distribution.^8^ ^,^ ^9^ ^,^ ^16^ For example, the carinae limiting the central depression of the scutellum is entirely black in specimens from Bolivia, Brazil, Costa Rica, Peru and Venezuela, while it is red or reddish in Colombia, Panama and Suriname insects.^8^ ^,^ ^16^ A traditional morphometric analysis of head capsules of individuals found in the domiciles from Bolivia (La Paz) versus sylvatic specimens deposited in the collection of the Natural History Museum of London (NHM), from Mexico, Ecuador and Panama (including the holotype of *P. rufotuberculatus*), showed a consistent variation of metric properties.^9^ Until now, no geometric morphometric approach had been applied.

As other hemipteran species, *Panstrongylus* genus presents holocentric chromosomes characterised by the absence of a primary constriction. Chromosomal analyses of eight out of the 14 *Panstrongylus* species reveal that this genus is variable in its diploid chromosome number (21, 23 or 24 chromosomes in males), including the number of autosomes (18 and 20), sex chromosome systems (X_1_X_2_Y and X_1_X_2_X_3_Y) and different amount of autosomal C-heterochromatin.^17^ Previous cytogenetic studies on *P. rufotuberculatus* were restricted to individuals from Colombia (Antioquia and Santander) showing a male diploid chromosome number of 23 chromosomes (20 autosomes plus X_1_X_2_Y) and C-heterochromatic regions on most autosomes.^18^

We examined the cytogenetic characteristics of *P. rufotuberculatus* individuals collected in several localities from three countries (Bolivia, Colombia and Ecuador). This material was also employed to address wing morphometrics analyses, which included an additional sample from French Guiana not analysed by cytogenetics. We determined, for the first time, the chromosomal position of major ribosomal loci (rDNA clusters) by fluorescence *in situ* hybridisation (FISH). This chromosomal trait is considered as species specific and very useful to distinguish chromosomally undifferentiated species, such as observed in several species complexes.^19^ ^,^ ^20^ ^,^ ^21^ In order to check the phylogenetic relationships of these individuals, sequences analyses of one nuclear (internal transcribed spacer 2 - ITS-2) and two mitochondrial DNA fragments (cytochrome b - *cyt b*- and cytochrome C oxidase subunit I - *coI*) were performed, including sequences of *P. rufotuberculatus* from different countries and others *Panstrongylus* spp. available in GenBank.

## MATERIALS AND METHODS

**Fig. 1:**
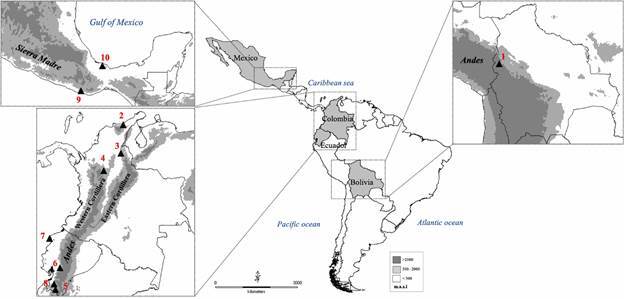
Latin American map showing the geographic location of the 10 sampling sites of *Panstrongylus rufotuberculatus* individuals studied by genetic markers in this paper. Site names and other details are given in Table I.

*Materials -* The specimens were identified according to morphological keys established in Lent and Wygodzinsky.^1^ Table I and Fig. 1 show the geographic origin and number of *P. rufotuberculatus* individuals studied by morphometric, cytogenetics and molecular analyses (including its GenBank accession numbers). Previously published cytogenetic data are also incorporated.^18^

**TABLE I:** Geographic origin of *Panstrongylus rufotuberculatus* specimens used in molecular analyses (ITS-2, *cyt b* and *coI*), cytogenetics (CYT) and morphometrics of wings (MM)

Country	Map number	Province/Department, Locality, Habitat	ITS-2	*cyt b*	*coI*	CYT	MM
Bolivia	1	La Paz, Muñecas, Ayata, Camata, I & P.	MZ647516	MZ661732	MZ643668, MZ643669	8	10M, 10F
Colombia North	2	La Guajira, Dibulla, Gumake, I & P.	ND	MZ661731	MZ643670, MZ643671	7	2M, 1F
	3	Norte de Santander, El Carmen, S.	AJ306546	ND	ND	5^*^	ND
Colombia Central	4	Antioquia, Amalfi & Yolombó, P & S	MZ647517	ND	MZ643672	6^*^	ND
Ecuador	5	Loja, Ardanza & La Ciénega, I.	MN505071-77 (7)	MN504825-30 (6)	MZ643673	5	2M, 9F
	6	Santo Domingo, Bella Vista, P	MN505078	ND	ND	2	ND
	7	Manabí, different localities, D, P & S.	MN505079-85 (7)	MN504831, MN504832	ND	ND	6M, 8F
	8	El Oro, Guayacán	AJ306545	ND	ND	ND	ND
Mexico	9	Oaxaca, Ciénaga Grande, P.	ND	ND	MZ643674	ND	ND
	10	Veracruz, Laguna Azul, San Andrés Tuxtla, S.	ND	NC042682	NC042682	ND	ND
French Guiana		Belizon	ND	ND	ND	ND	19M
		TOTAL INDIVIDUALS	19	11	8	33	39M, 28F

I: intradomiciliary; P: peridomiciliary; S: Sylvatic; M: males; F: females; ND: not determined. *: chromosome data from Crossa et al.^18^ ^)^ In bold: accession numbers of sequences obtained in this study

In our phylogenetic analyses, we used all *Panstrongylus* sequences available in GenBank, in addition to sequences obtained by us. However, several ITS-2 and *cyt b* sequences identified as *P. chinai*, *P. howardi* and *P. rufotuberculatus*, submitted in GenBank in 2014 by Sempertegui-Sosa et al., were not included (Unpublished data). Barnabé et al.^13^ recognised, in several of these sequences, labeling errors in their species identification. Since *Panstrongylus* species belong to the North American Triatomini lineage, other species from this lineage were also included, as well as several species from the South American lineage as out-groups. Since there were few *coI* sequences available in GenBank for the fragment employed in the present paper, we have sequenced individuals of other *Panstrongylus* species: *P. chinai* (MZ643675); *P. lignarius/herreri* (MZ643676); *P. geniculatus* (MZ643678, MZ643679) and *P. tupynambai* (MZ643677).

*Morphometric study* - The wings pictures assembled for these morphometric studies were provided by different authors (TC, AV, JPD and two others cited in the acknowledgments). Since the pictures from Bolivia and Colombia did not harbor a size scale, our statistical analyses of the total sample were restricted to shape comparisons only, without including size analyses.

**Fig. 2:**
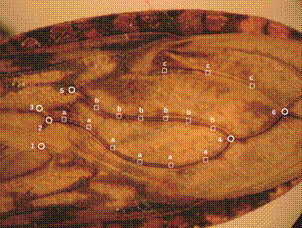
landmarks and semilandmarks of the wing venation in *Panstrongylus rufotuberculatus*. Numbers correspond to true landmarks type I (LM I) and letters to semilandmarks. The semilandmarks are partly capturing the shape of three veins: the anal vein (group “a”), the cubital vein (group “b”) and the median vein (group “c”). Their positions were computed depending on the position of two flanking landmarks.

**Fig. 3:**
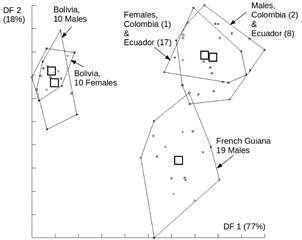
factor map of the two first discriminant factors (DF 1, horizontal axis and DF 2, vertical axis) derived from first nine principal components which together represented 73% of the total shape variation in *Panstrongylus rufotuberculatus*. The contribution of each DF to the total discriminant space is indicated between brackets. Bolivia is completely distinguished on DF1, French Guiana is distinguished on DF2.

Six landmarks were selected on the wings in addition to 14 semilandmarks that were used to capture the curved lines of veins between landmarks (Fig. 2). All landmarks and semilandmarks were submitted to partial Procrustes superimposition (GPA),^22^ and semilandmarks then subjected to sliding procedure.^23^ The tangent space projections of 67 individuals were used as input for a principal component analysis (PCA). The discriminant analysis used the nine first PCA as input, and was illustrated by the factor map of first and second discriminant factor (Fig. 3). This analysis compared males, females and countries as separated groups. Because of the very small samples from Colombia, this country was mixed with the neighbor country Ecuador.

*Cytogenetic studies* - We analysed 33 *P. rufotuberculatus* bugs whose geographical origin is detailed in Table I. Gonads were removed from adult insects and fixed in ethanol-acetic acid (3:1). C-banding and fluorescent in situ hybridisation (FISH) using as a probe an 18S rDNA fragment (included within 45S rDNA clusters) were carried out on chromosome preparations obtained by the squash method.^18^ ^,^ ^19^ Slides were examined under a Nikon Eclipse 80i microscope and the images were obtained with a DS-5Mc-U2 digital camera. For each specimen, at least 20 cells were analysed to determine the chromosome number, the C-banding pattern and the chromosomal location of the 45S rDNA clusters. The C-banding pattern of *P. rufotuberculatus* was previously described on individuals from Colombia (Antioquia and Santander).^18^ In this paper, we included more individuals from the previously reported populations, as well as from other localities in Colombia (La Guajira and Yolombó), Ecuador (Loja and Santo Domingo) and Bolivia (La Paz). The chromosomal locations of 45S ribosomal clusters were determined by FISH in *P. rufotuberculatus* individuals and described here for the first time. To elucidate the possible origin of the sex chromosome polymorphism, we estimate the relative length (in percent) of the X chromosome(s) in relation to the chromosome complement total length. Images were analysed using the Nikon Nis Elements 3.1. Advanced Software. We analysed 100 metaphase I and II images from three individuals from each sex chromosome system.

*DNA extraction and sequencing* - Bugs legs, stored in 70% ethanol, were used for DNA extraction using standard phenol-chloroform technique. A 210 bp ITS-2 fragment was amplified by PCR using the 5.8T and 28T primers,^4^ a 600 bp fragment of *coI* was amplified with ACO and COIb primers,^24^ and a 419 bp fragment of *cyt b* with the primers CYTB7432F and CYTB7433R.^25^ PCR products were Sanger-sequenced by Macrogen Inc. (Korea). Forward and reverse sequences were aligned using Bioedit v.7.0.9.

*Genetic diversity and phylogenetic analyses* - Considering that ITS-2 nuclear sequences have wide size differences among species, gaps (indels) were included as informative characters in sequence-based and alignment analyses^26^ and added as an additional block into the data file using FastGap v.1.2 software (http://www.aubot.dk/FastGap_home.htm).

For the three molecular markers, MAFFT v7.453 was employed to align sequences. Genetic distances for mitochondrial markers were calculated using APE package v5.4.1^27^ in R v3.6.1, using the Kimura 2-parameter substitution model (K2-p). The best fitting substitution models for the mitochondrial genes were estimated using Smart Model Selection and jModelTest v.2.1.10^28^ for the nuclear ITS-2. Decisions were taken under Bayesian information criteria (BIC).

Maximum likelihood (ML) phylogenetic trees were obtained using PhyML v3.^29^ Bootstrap values were estimated from 1000 replicates. In order to include indels information for ITS-2 sequences, a Bayesian phylogenetic tree was inferred with MrBayes v.3.2.7a.^30^ The posterior probability of the phylogenetic tree was estimated by Metropolis-coupled Markov chain Monte Carlo (MCMCMC). The MCMCMC settings consisted of two simultaneous, independent runs with four chains each (with default heating temperature 0.1), which were run for 10×6 generations and sampled every 1000 generations with a 25% burn-in. Convergence was assessed using the average standard deviation of split frequency values (< 0.01) and by the Effective Sample Size (ESS > 200).

## RESULTS

*Morphometric variation (* *Fig. 3* *)* - On the discriminant space derived from shape variables, males and females were grouped together as expected. The first discriminant factor (DF) represented 77% of the total shape variation among samples (Fig. 3). On this first axis, males and females from Bolivia constituted a completely separated group. The second DF represented 18% of the total shape variation, separating the Guiana sample from the others, but not completely from the Colombia-Ecuador sample. The remaining 2 DF represented only 4% of the total variation and were not presented here.

**Fig. 4:**
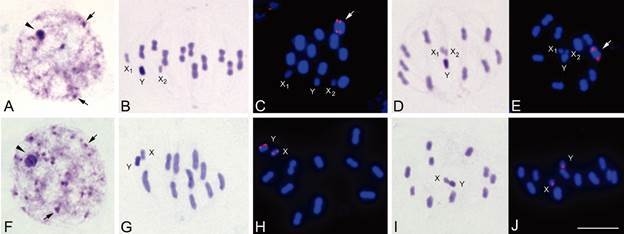
male meiosis in *Panstrongylus rufotuberculatus*. Top row (A-E): *P. rufotuberculatus* from Colombia and Ecuador named cytotype 1 (2n = 23 = 20A + X_1_X_2_Y) showing two X chromosomes and 45S ribosomal clusters on one autosome. Bottom row (F-J): *P. rufotuberculatus* from Bolivia named cytotype 2 (2n = 22 = 20A + XY) showing only one X chromosome and ribosomal clusters in X and Y chromosomes. C-banding: A, B, D, F, G and I. Fluorescent *in situ* hybridization with 18S ribosomal DNA probe: C, E, H and J. Chromosomes in blue (DAPI stain) and ribosomal signals (45S rDNA clusters) in red on one autosome (arrows in C and E) or on the X and Y chromosomes (H and J). (A and G): First meiotic prophases showing C-dots dispersed in the nuclei. First and second meiotic metaphases (B, C, G, H and D, E, I, J respectively). Bar = 10 μm.

*C-banding and fluorescence in situ hybridisation (* *Fig. 4* *)* - Chromosomal analyses of 33 *P. rufotuberculatus* individuals from Bolivia, Colombia and Ecuador identified two chromosomal groups, also called patterns or cytotypes, with differences in the number of sex chromosomes and in the chromosomal location of 45S ribosomal clusters. Both cytotypes presented autosomal C-heterochromatin located at one or both chromosomal ends in almost all autosomal pairs.

Cytotype 1: Individuals from Colombian and Ecuadorian populations (Fig. 4A-E, top row,). Male individuals presented a diploid number (2n) of 23 chromosomes, with 10 autosomal pairs plus three sex chromosomes: X_1_X_2_Y. Females have 2n = 24 (20 autosomes plus X_1_X_2_X_1_X_2_). Early meiotic prophase was characterised by the presence of a largest heterochromatic chromocenter constituted by the association of the sex chromosomes (Fig. 4A, arrowhead). Furthermore, several heterochromatic dots were scattered throughout the nucleus (Fig. 4A, arrows), corresponding with terminal C-regions of autosomal pairs. Autosomal chromosomes showed scarce variation regarding their size (Fig. 4B-E). The Y sex chromosome was middle sized and totally C-heterochromatic, while X_1_ and X_2_ chromosomes were the smallest of the complement and had an intermediate staining (Fig. 4B,D). In metaphase II, the three sex chromosomes disposed as a pseudotrivalent, in the center of an autosomal ring-like shape. FISH results indicated that 45S rDNA clusters were located on the largest autosomal pair (Fig. 4C, E, arrows).

Cytotype 2: Individuals from Bolivian population (Fig. 4F-J, bottom row). All individuals presented a diploid number (2n) of 22 chromosomes, with 10 autosomal pairs plus two sex chromosomes (XY in males and XX in females). Autosomal C-heterochromatin distribution was similar to what was observed in individuals from Colombia and Ecuador (Fig. 4A, F). In addition, the Y sex chromosome was middle sized and totally heterochromatic. The X chromosome was similar in size to the Y chromosome (Fig. 4G-I) with an intermediate staining. During metaphase II, the X and Y chromosomes formed a pseudobivalent (Fig. 4I). The 45S rDNA clusters were situated on both sex chromosomes (X and Y) (Fig. 4H, J).

To clarify the origin of the sex chromosome polymorphism, we measured the average length of the X chromosomes over the total length of the chromosome complement. In Bolivian male individuals, presenting XY sex chromosomes, the mean length of the X chromosome represented 5.98 ± 0.17%. In the individuals from Colombian and Ecuadorian populations, the sum of the X_1_ and X_2_ chromosomes average lengths represented 5.87 ± 0.15%.

**Fig. 5:**
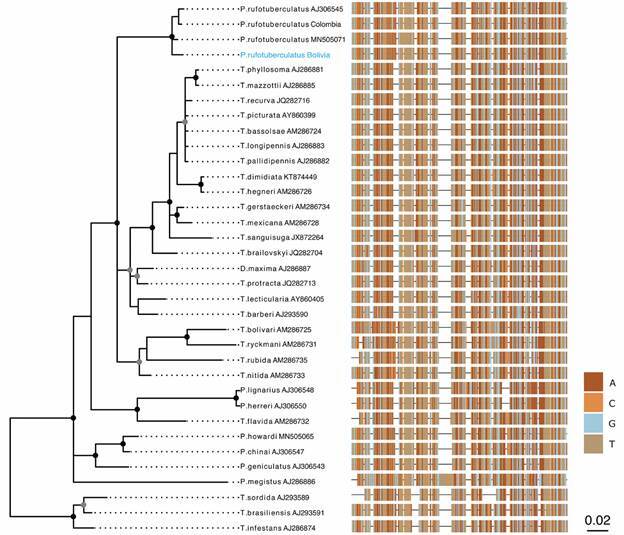
Bayesian phylogenetic tree obtained from ITS-2 fragments including the sequences of the six *Panstrongylus* species available in GenBank (*P. lignarius* and *P. herreri* counted as one species). The *Panstrongylus* genus is paraphyletic; with *P. rufotuberculatus* clade clearly separated from the other *Panstrongylus* species. The 19 individuals of *P. rufotuberculatus*, showed as four haplotypes, are clustered in one clade, including *P. rufotuberculatus* from Bolivia. Circles represent statistical support obtained through posterior probability, gray circles denote above 0.75 and black circles above 0.9. DNA sequence alignment is represented at the right panel, where each color represents a different DNA base. Accession numbers of *P. rufotuberculatus* from Bolivia and Colombia are MZ647516 and MZ647517 respectively.

*Nuclear ITS-2 (* *Fig. 5* *)* - The best fitted model was the HYK + G and selected for the construction of the ITS-2 Bayesian tree. This phylogenetic tree included sequences of the five *Panstrongylus* species together with 19 sequences of *P. rufotuberculatus* available in GenBank, two of them sequenced in this study (Table I). Identical sequences were grouped in the same haplotype, so only one was represented in the tree [Supplementary data (Table)]. Since *Panstrongylus* species belong to the North American lineage, other triatomine species from this lineage were also included. South American lineage species are considered as out-groups. This analysis showed that the *Panstrongylus* genus is clearly paraphyletic. All *P. rufotuberculatus* sequences (four haplotypes representing 19 sequences) are grouped together in one clade with high statistical support, and also separated from the remaining five *Panstrongylus* species.

**Fig. 6:**
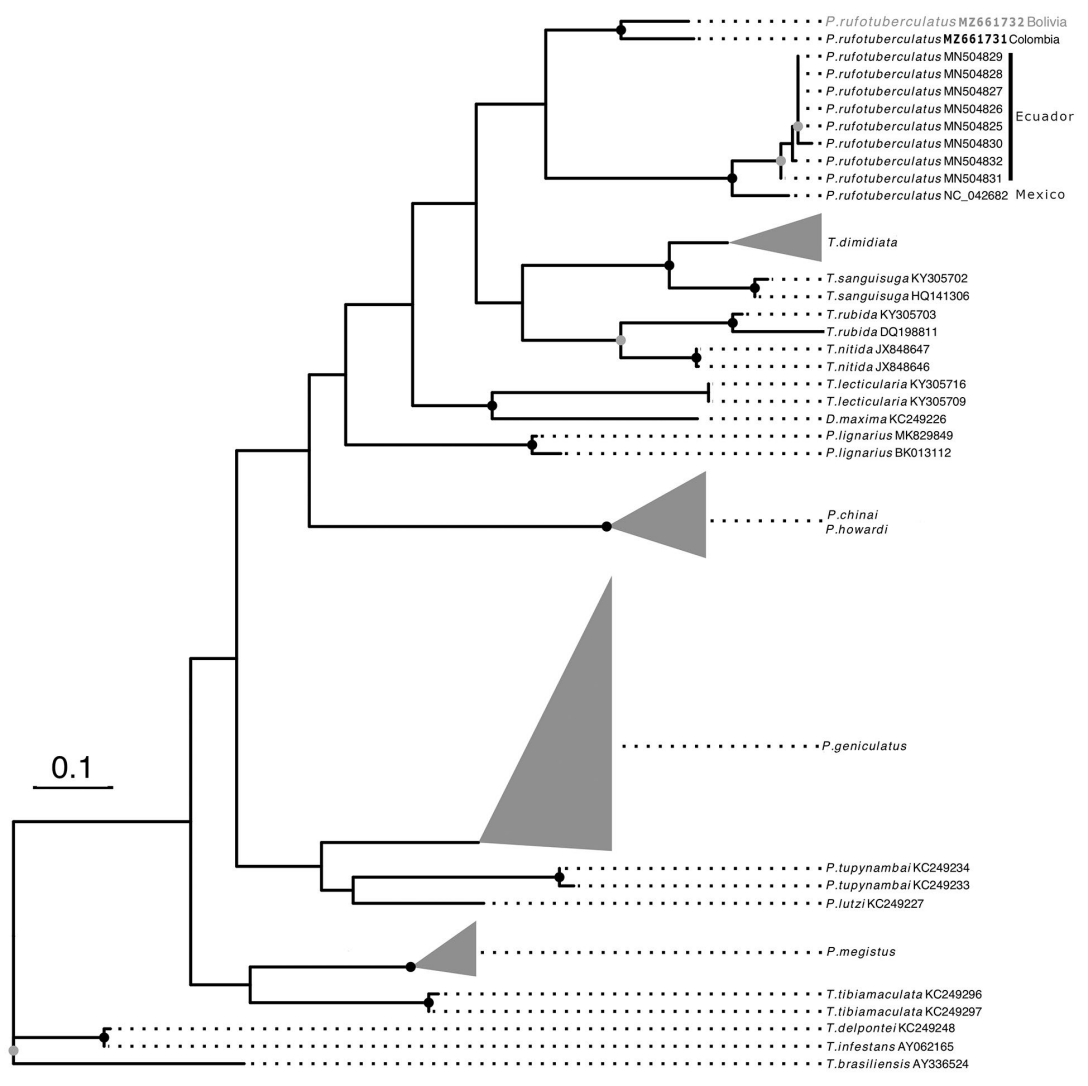
collapsed maximum likelihood phylogenetic tree obtained from cytochrome b (*cyt b*) fragment, including all sequences of *Panstrongylus* species available in GenBank. The paraphyly of the *Panstrongylus* genus is clearly shown. The 11 individuals recognized as *P. rufotuberculatus* were grouped into two well supported clades, clearly separated from the remaining seven *Panstrongylus* species. The individual from Bolivia (in blue) is closely related to *P. rufotuberculatus* from Colombia (La Guajira). Accession numbers of sequences presented herein are depicted in bold. Gray circles denote bootstraps support above 0.75 and black circles above 0.9. Uncollapsed tree could be seen in Supplementary data (Figure).

*Mitochondrial cyt b fragment (* *Fig. 6* *)* - Maximum likelihood (HKY85+G+I) analysis using *cyt b* fragment sequences of seven *Panstrongylus* species plus *P. rufotuberculatus* is shown in Fig. 6 (collapsed tree). This phylogenetic analysis depicted the genus *Panstrongylus* as a paraphyletic clade. The 11 sequences identified as *P. rufotuberculatus* were grouped into two well supported clades. One of them includes the sequences from Bolivia and Colombia (La Guajira) and the other one the remaining sequences from Ecuador and Mexico. Uncollapsed tree could be seen in Supplementary data (Figure).

**Fig. 7:**
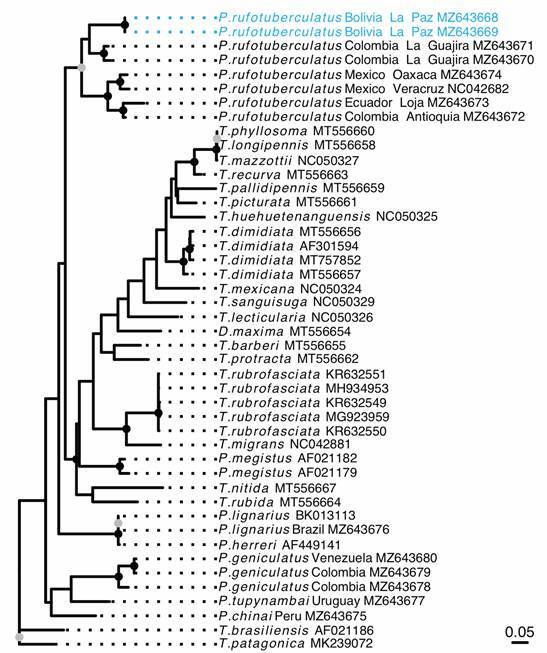
maximum likelihood phylogenetic tree obtained from *cytochrome C oxidase subunit I* (*coI*) fragment from six *Panstrongylus* species, including all other triatomine species available in GenBank. The genus *Panstrongylus* is clearly paraphyletic. The eight individuals of *P. rufotuberculatus* are grouped in the same clade, clearly separated from the remaining five *Panstrongylus* species. Depicted in blue are *P. rufotuberculatus* from Bolivia. Gray circles denote bootstraps support above 0.75 and black circles above 0.9.

*Mitochondrial coI fragment (* *Fig. 7* *)* - The GTR+G+I substitution model was selected for the construction of the *coI* ML tree. This analysis included five *Panstrongylus* species plus sequences of *P. rufotuberculatus*. This genetic marker also supports the paraphyly of *Panstrongylus*. All individuals (eight) initially identified as *P. rufotuberculatus* are closely related to each other constituting a clearly separated clade from the remaining *Panstrongylus* species. Within this clade, it is possible to identify four well supported subclades: (a) Mexican individuals from Oaxaca and Veracruz, (b) Ecuadorian and Central Colombia (Antioquia) individuals, (c) North Colombia (La Guajira), and (d) Bolivian specimens. The specimens from Bolivia appear as a sister group of *P. rufotuberculatus* from Colombia (La Guajira).

Average genetic distances (K2-p) among the four *P. rufotuberculatus* groups vary between 10.0% to 19.7% for *cyt b* and from 9.0% to 15.8% for *coI* fragments. Comparing *P. rufotuberculatus* from Bolivia with the other groups, the average genetic distances are close to the highest values, varying between 10.7% and 18.7% for *cyt b* and 10.6% and 15.8% for *coI* fragments. For both markers, the closest group to Bolivian individuals is the one from North Colombia (La Guajira): K2-p of 10.7% for *cyt b* and 10.6% for *coI* (Table II).

**TABLE II:** Mitochondrial DNA sequence differentiation among four *Panstrongylus rufotuberculatus* geographic groups represented in Table I. Mean Kimura 2-parameter (K2-p) distances (in percentages) computed with *cyt b* (above diagonal) and *coI* (below diagonal) partial sequences; mean within-group distances (*cyt b* / *coI*) are on the diagonal

Cyt b / coI	Mexico	Ecuador + Central Colombia	North Colombia (Guajira)	Bolivia (La Paz)
Mexico	0 / 3.93	10.04	19.72	18.75
Ecuador + Central Colombia	9.00	1.56 / 5.42	16.65	18.06
North Colombia (Guajira)	12.78	12.30	0 / 2.64	10.71
Bolivia, La Paz	14.02	15.81	10.58	0 / 0

## DISCUSSION

*Wing venation patterns* - Due to the incomplete information about the wings size, no comparisons of size were possible, and its possible effect on the shape variables as extracted from our landmarks and semilandmarks remains unknown. Our work hypothesis considers that the possible allometric effect on shape variables did not bias the global comparison.

Shape as disclosed by the geometric morphometrics approach is a polygenic character^31^ and most of its variation has been attributed to genetic drift rather than to environmental conditions.^32^ For this reason, the shape-based discriminant space is frequently reminiscent of the geographic map, as in our case here (Fig. 3).

The clear cut shape-based discrimination obtained between the Bolivian sample and the remaining ones parallels the separate position of the Bolivian origin in the morphospace of the head capsule.^9^ Considering the wide geographic sample of *P. rufotuberculatus* individuals, the observed morphometric variation pattern is likely to be the reflection of genetic drift. However, the morphometric approach by itself cannot tell us whether it is due to a spatial or biological isolation.

*Sex chromosome polymorphism in Heteroptera* - In Triatominae as well as in Reduviidae, it is generally agreed that XX/XY is the ancestral sex chromosome system. Sex mechanisms with multiple X or Y chromosomes are believed to be most probably originated through fragmentation(s) of the ancestral X and Y chromosome, respectively.^33^ Intraspecific variation in the number of sex chromosomes (consider as polymorphisms) are very uncommon. It has been detected in only ten of the more than 1,000 heteropteran species analysed so far.^33^ ^,^ ^34^ ^,^ ^35^ Almost all of these variations were due to the fragmentation of the X chromosome, which was supported both by their meiotic chromosomal behavior and by the size comparison of the sex chromosomes between the individuals with simple system (XY) and those with multiple systems (XnY).^34^ ^,^ ^35^ Up to now, intraspecific variation in the number of sex chromosomes has not been reported in the Triatominae subfamily.

Almost all species from the North American Triatomini lineage (38 out of 41), which includes all *Panstrongylus* species, presented multiple X chromosomes. According to Panzera et al.,^17^ the X_1_X_2_Y chromosome sex system is considered as the ancestral one in this lineage. Therefore, the XY mechanism observed in Bolivian individuals could represent a derived character originated by a fusion of the ancestral X_1_ and X_2_ chromosomes. The X chromosome relative length in Bolivian *P. rufotuberculatus* was significantly similar to the sum of chromosomes X_1_ and X_2_ of the individuals from Ecuador and Colombia. Hence, the fusion of the X_1_ and X_2_ chromosomes is the most likely explanation for the emergence of the XY system in *P. rufotuberculatus* from Bolivia.

*Location of 45S ribosomal clusters* - Another chromosome variation found within *P. rufotuberculatus* was the chromosomal position of the major ribosomal DNA loci. In the Bolivian individuals, the rDNA clusters were located on both sex chromosomes (Fig. 4F-J, bottom row), while in individuals from Colombia and Ecuador they were on one autosomal pair (Fig. 4A-E, top row).

The analysis of 92 triatomine species so far, revealed that the chromosomal position of the 45S ribosomal clusters was a species specific character.^21^ Intraspecific variation in the rDNA clusters location is an exceptional event within Hemiptera, reported only in one triatomine species: *T. infestans*.^19^ ^,^ ^36^ Another apparently intraspecific variation reported in *Rhodnius ecuadoriensis* from Peru and Ecuador by Pita et al.^37^ turned out to be explained by the existence of different species.^38^

In the Triatomini tribe, the location of rDNA clusters on one autosomal pair is by far the most frequent pattern and is considered as the ancestral character for this group.^17^ ^,^ ^21^ This trend is more acute in the North American lineage, since the autosomal rDNA location is highly conserved including all *Panstrongylus* species analysed hitherto.^21^ Therefore, the sex chromosome location pattern observed in *P. rufotuberculatus* from Bolivia is a very atypical event and should be considered as an apomorphic character. The movement of the ribosomal clusters from autosomes to both sex chromosomes is probably due to transposition events or ectopic recombination, as observed in other triatomine species.^17^ ^,^ ^19^ ^,^ ^21^ The mobilisation of rDNA loci from autosomes to sex chromosomes alters the genetic recombination of the involved chromosome regions. The differences in genetic recombination rates among autosomes and sex chromosomes are even more extreme in triatomines considering the achiasmatic nature of sex chromosomes.^17^ ^,^ ^19^ ^,^ ^21^ ^,^ ^39^ As a result of the reduced recombination, genetic barriers to gene flow could arise rapidly between populations with different sex chromosome systems and location of ribosomal clusters, leading to their divergence and speciation. This was suggested for other insect groups such as Coleoptera and Lepidoptera.^40^ ^,^ ^41^ As gene flow is more restricted between sex chromosomes than autosomes, sex linked genes are particularly efficient to produce post-zygotic barriers^42^ and therefore rearrangements involving sex chromosomes can be particularly effective as isolation mechanisms.

Most likely, crosses between *P. rufotuberculatus* individuals with different cytotypes could give rise to an adult progeny, which will include individuals with different numbers of X chromosomes and chromosomes with ribosomal loci. As a consequence of chromosomal pairing and segregation during both meiotic divisions, this hybrid progeny will produce a number of unbalanced gametes, both in the number of X chromosomes and of chromosomes carrying ribosomal loci, resulting in reproductive disadvantages. This negative effect is exacerbated by the changes in the number of X chromosomes because they alter the sex determination control and the gene regulation. A similar effect was described in coleopteran species.^40^

We propose that the variability in the number of X chromosomes and rDNA position observed in *P. rufotuberculatus* populations reflects chromosomal rearrangements leading to reproductive incompatibility between both cytotypes. Hence, it is likely that they do not represent intraspecific variability.

*Molecular markers* - In concordance with previous phylogenetic analyses,^4^ ^,^ ^5^ ^,^ ^6^ ^,^ ^43^ our results support the paraphyletic nature of the *Panstrongylus* genus and their inclusion within North American Triatomini lineage (Figs 5-7). Furthermore, previous reports and the here obtained results also showed that *P. rufotuberculatus* specimens constitute always a separate clade from the remaining *Panstrongylus* species. Despite the low number of individuals here analysed (38 for the three molecular markers), phylogenetic trees also showed that all individuals morphologically identified as *P. rufotuberculatus,* including those from Bolivia, are closely related. In conclusion, despite its important chromosomal differences, *P. rufotuberculatus* from Bolivia seems to be a sister group to the remaining *P. rufotuberculatus* individuals of other countries.

The average genetic distances (K2-p) of *P. rufotuberculatus* from Bolivia to others *P. rufotuberculatus* varies between 10.7% to 18.7% and 10.6% to 15.8% for *cyt b* and *coI* fragments respectively (Table II). The closest individuals to the Bolivian samples are those from La Guajira (North Colombia) with 10.7% and 10.6% K2-p distances respectively. The last ones present the regular chromosomal cytotype (Fig. 4). For *cyt b* gene, this distance was much greater than the cut-off value of 7.5% proposed by Monteiro et al*.* ^44^ to validate distinct triatomine species. For example, within the closely related species of dimidiata complex (*T. dimidiata and those later described as T. mopan* ^45^ *and T. huehuetenanguensis* ^46^), the *cyt b* sequences diverged from 8.0% to 15.5%.^47^ Furthermore, *Panstrongylus* sister species such as *P. chinai* and *P. howardi* presented a K2-p distance of 7.8%.^48^ For *coI* sequences, the value of 10.6% observed between *P. rufotuberculatus* from Bolivia and La Guajira was also significantly higher than those reported for other closely related triatomine species, as 7.1% between *Mepraia spinolai* and *M. gajardoi,* ^24^ or 5.3% between *T. sordida* and the recently described species *T. rosai*.^49^ In summary, the great genetic differentiation observed between the two chromosomal cytotypes of *P. rufotuberculatus*, either by *cyt b* and *coI* fragments, suggests that both groups should be considered as distinct species.

Within *P. rufotuberculatus*, both mitochondrial markers identified four groups: Bolivia, Mexico, North Colombia, and Ecuador plus central Colombia (Table II, Figs 1, 5-7). Unexpectedly, the genetic K2-p distances observed between these four groups are similar to those observed between different species. This would suggest that *P. rufotuberculatus* is most likely a complex of several taxa (Table II). Therefore, it is essential to analyse a greater number of individuals to confirm these high genetic divergences. Unfortunately, chromosomal data from the Mexican group is unavailable at the moment.

*Evolutionary origin of P. rufotuberculatus from Bolivia* - The close relationship obtained in the phylogenetic analyses and the external morphology similarities suggest that *P. rufotuberculatus* from Bolivia is a sister group of the remaining *P. rufotuberculatus* from other countries (Figs 4-6). Variations in the number of X chromosomes and the rDNA loci chromosomal location of *P. rufotuberculatus* from Bolivia are most likely explained by at least two different chromosomal rearrangements. Our hypothesis implies an ancestor with a multiple sex system (X_1_X_2_Y) and ribosomal clusters in one autosomal pair, similar to most *Panstrongylus* species (including *P. rufotuberculatus* from Colombia and Ecuador). In this ancestor, the first rearrangement that should have occurred was the fusion of the X_1_ and X_2_ chromosomes, resulting in a single fused X chromosome. Subsequently, the complete mobilisation of the ribosomal loci from one autosome pair to the newly fused X chromosome may have occurred, followed by another transfer (partial) of rDNA clusters from the X chromosome to the Y chromosome. Considering the regular meiotic segregation of sex chromosomes in Bolivian individuals, and the fact that heterozygous individuals for each chromosomal marker were not detected, it is very likely that both rearrangements occurred a long time ago. This could be supported by the significant genetic distances observed among *P. rufotuberculatus* from Bolivia and its sister groups (Table II).

The present paper reports, for the first time, an extensive morphometric and genetic differentiation within *Panstrongylus rufotuberculatus*. Important chromosomal differences, that would prevent genetic exchange between both chromosomal groups, are here revealed. This evidence is supported by high values of genetic distances and consistent morphometric differences between the groups. Deeper and widely molecular analyses would help to confirm genetic differentiation observed among *P. rufotuberculatus* from different countries.

All the *P. rufotuberculatus* material from Bolivia here analysed, was collected in 2004 and 2006 and belongs to a single locality (La Paz, Muñecas, Camata), both from domiciliary and peridomiciliary environments. However, the geographical distribution of *P. rufotuberculatus* in Bolivia is wider, since it has already been described in other provinces of La Paz Department (Nor Yungas, Sud Yungas, Caranavi, Inquisivi), associated with anthropic structures, particularly intradomiciles.^8^ ^,^ ^9^ ^,^ ^10^

A detailed morphological examination and other biological aspects of *P. rufotuberculatus* from Bolivia are now needed to determine external differences that allow its taxonomic recognition as well as for its formal description as a separated species.
