# Supplementary material for: Multidisciplinary approach detects speciation within the kissing bug Panstrongylus rufotuberculatus populations (Hemiptera, Heteroptera, Reduviidae)
Source: Mem Inst Oswaldo Cruz. 2022 Feb 2;116:e210259. doi: 10.1590/0074-02760210259 (PMC8815762; doi:10.1590/0074-02760210259)
Supplement: Supplementary file 1 [file 1678-8060-mioc-116-e210259-s.pdf]

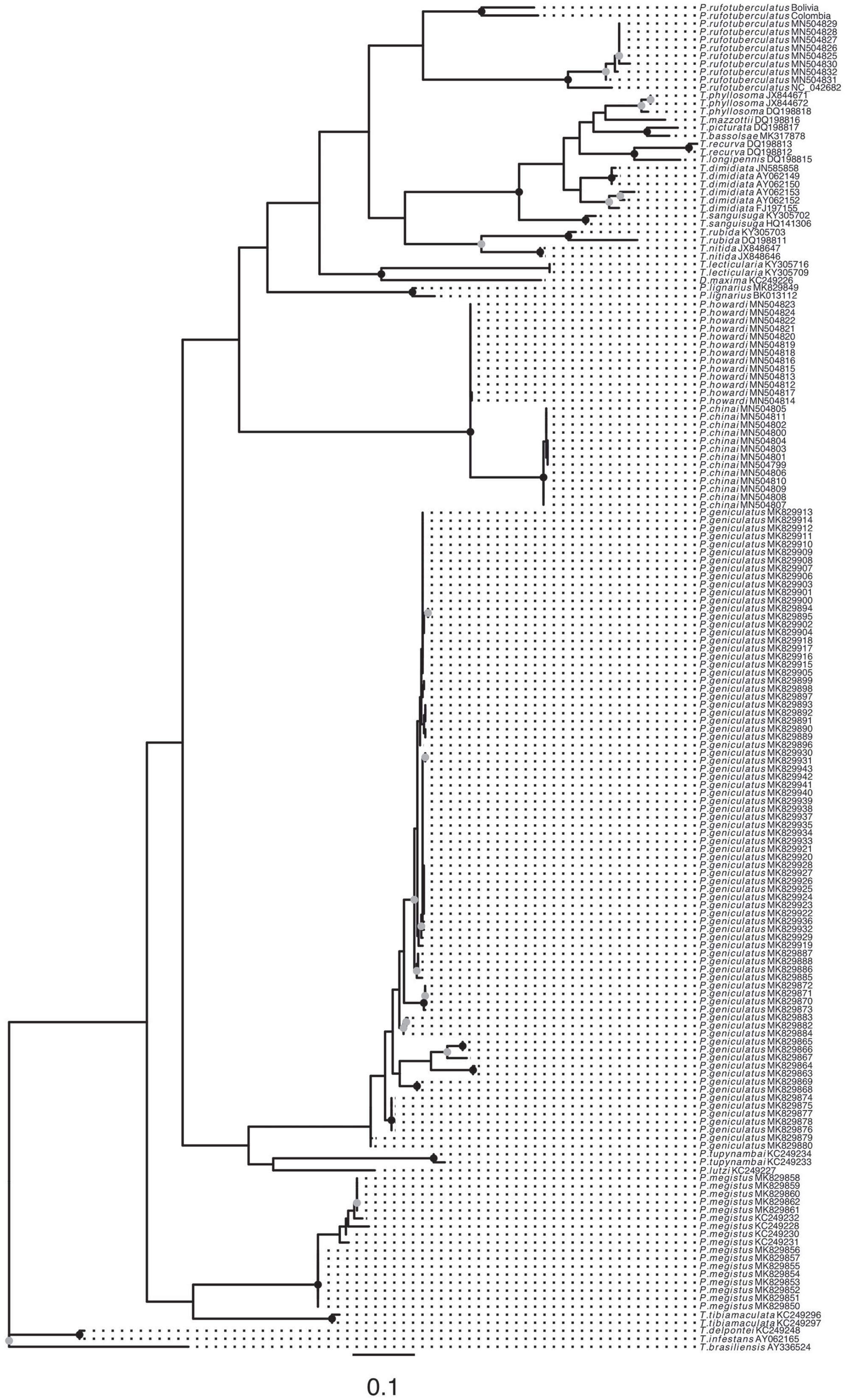

Uncollapsed maximum likelihood phylogenetic tree obtained from cytochrome b (cyt b) fragment, including all sequences of *Panstrongylus* species available in GenBank (collapsed tree shown in Fig. 6). The 11 individuals recognised as *P. rufotuberculatus* were grouped into two well supported clades, clearly separated from the remaining seven *Panstrongylus* species. Gray circles denote bootstraps support above 0.75 and black circles above 0.9.

cc BY

TABLE

Gen Bank Accession Numbers ITS-2 fragments used in Fig. 5

Species	Number of sequences	GenBank Acc. Numbers (bold sequences used in fig. 5)	Geographic origin. More details Table I
Panstrongylus			
P. rufotuberculatus	1	**MZ647516**	Bolivia (La Paz)
	6	**MZ647517;** MN505078-81, MN505085	Colombia (Guajira); Ecuador (Manabí + S. Domingo)
	2	**AJ306545**- 46	Colombia (Norte Santander), Ecuador (El Oro)
	10	**MN505071**-77, MN505082-84	Ecuador (Loja + Manabí)
P. chinai	10	**AJ306547**, MN505056-64	
P. howardi	6	**MN505065**-70	
P. geniculatus	2	**AJ306543**-44	
*P. herreri**	2	**AJ306550**-51	
*P. lignarius**	2	**AJ306548**-49	
P. megistus	27	**AJ286886**, AJ306542, HF678452-60, HF678462-77	
Triatoma			
T. hegneri	2	**AM286726**-27	
T. mexicana	2	**AM286728**, JQ282710	
T. gerstaeckeri	2	**AM286734**, JQ282707	
T. pallidipennis	6	**AJ286882**, AM286729-30, AY860395, AY860403, JX872262	
T. longipennis	4	**AJ286883**, AY860397-98, KC698909	
T. barberi	2	**AJ293590**, JX872261	
T. bassolsae	5	**AM286724**, AY860394, AY860400-02	
T. bolivari	2	**AM286725**, JQ282701	
T. brailovskyi	2	**JQ282704**, JQ282706	
T. brasiliensis	1	**AJ293591**	
T. dimidiata	2	**KT874449**, MN505086	
T. flavida	1	**AM286732**	
T. infestans	1	**AJ286874**	
T. lecticularia	3	**AY860405**-07	
T. mazzottii	4	**AJ286885**, AY860392-93, KC698911	
T. nitida	2	**AM286733**, JX872260	
T. phyllosoma	17	**AJ286881**, HQ185165-68, HQ185170/74/78/80/82-84/87-90, KC698912	
T. picturata	2	**AY860399**, AY860404	
T. protracta	4	**JQ282713**-15, JX872263	
T. recurva	1	**JQ282716**	
T. rubida	4	**AM286735**, AY860389-91	
T. ryckmani	2	**AM286731**, JX872266	
T. sanguisuga	4	**JX872264**-65, JX890270, KF142511	
T. sordida	1	**AJ293589**	
Dipetalogaster			
D. maxima	1	**AJ286887**	

*: *Panstrongylus herreri* and *P. lignarius* are considered the same species.
